# Update in myopia and treatment strategy of atropine use in myopia control

**DOI:** 10.1038/s41433-018-0139-7

**Published:** 2018-06-11

**Authors:** Pei-Chang Wu, Meng-Ni Chuang, Jessy Choi, Huan Chen, Grace Wu, Kyoko Ohno-Matsui, Jost B Jonas, Chui Ming Gemmy Cheung

**Affiliations:** 1 Department of Ophthalmology, Kaohsiung Chang Gung Memorial Hospital and Chang Gung University College of Medicine, Kaohsiung, Taiwan; 2 Department of Ophthalmology, Sheffield Children Hospital NHS Foundation Trust and Sheffield Teaching Hospital NHS Foundation Trust, Sheffield, UK; 3 Department of Ophthalmology, Peking Union Medical College Hospital, Beijing, China; 4 Singapore Eye Research Institutes, National University of Singapore, Singapore, Singapore; 5 Department of Ophthalmology and Visual Science, Tokyo Medical and Dental University, Tokyo, Japan; 6 Department of Ophthalmology, Medical Faculty Mannheim of the Ruprecht-Karls-University of Heidelberg, Mannheim, Germany

**Keywords:** Scleral diseases, Refractive errors

## Abstract

The prevalence of myopia is increasing globally. Complications of myopia are associated with huge economic and social costs. It is believed that high myopia in adulthood can be traced back to school age onset myopia. Therefore, it is crucial and urgent to implement effective measures of myopia control, which may include preventing myopia onset as well as retarding myopia progression in school age children. The mechanism of myopia is still poorly understood. There are some evidences to suggest excessive expansion of Bruch’s membrane, possibly in response to peripheral hyperopic defocus, and it may be one of the mechanisms leading to the uncontrolled axial elongation of the globe. Atropine is currently the most effective therapy for myopia control. Recent clinical trials demonstrated low-dose atropine eye drops such as 0.01% resulted in retardation of myopia progression, with significantly less side effects compared to higher concentration preparation. However, there remain a proportion of patients who are poor responders, in whom the optimal management remains unclear. Proposed strategies include stepwise increase of atropine dosing, and a combination of low-dose atropine with increase outdoor time. This review will focus on the current understanding of epidemiology, pathophysiology in myopia and highlight recent clinical trials using atropine in the school-aged children, as well as the treatment strategy in clinical implementation in hyperopic, pre-myopic and myopic children.

## 摘要

全球近视的患病率正在急剧上升, 且近视的并发症消耗巨大的社会和财政支出。人们认为, 成年后的高度近视由学龄期发生的近视而来。因此, 实施近视控制的有效措施至关重要, 措施包括预防近视产生以及延缓学龄儿童的近视发展。目前, 然而关于近视的致病机制仍然知之甚少。一些证据表明Bruch膜的过度扩张, 可能引起了周边远视离焦, 并且它可能是导致眼球发生不可控制的眼轴伸长的机制之一。阿托品是目前控制近视最有效的方法。最近的临床试验表明, 与高浓度制剂相比, 低剂量阿托品滴眼液如0.01% 可以延缓近视发展, 副作用明显减少。然而, 仍然有一部分患者的反应较差, 其中最佳处理仍不明确。已提出的临床策略包括逐步增加阿托品给药频率, 以及低剂量阿托品结合增加室外活动时间。本综述将重点介绍目前近视在流行病学、病理生理学中的研究, 以及最近在学龄儿童中使用阿托品的临床试验和临床实施的远视、近视、近视儿童的治疗策略。

Myopia is the most common eye disorder worldwide, but it is often misregarded as merely a refractive error that can simply be corrected by spectacles, contact lenses, or refractive surgery. As a matter of fact, high myopia is often associated with an increased risk of a range of serious ocular complications, which may result in irreversible vision loss. The World Health Organization (WHO) recently defined “high myopia” as −5 Diopter (D) or greater, which is associated with increased risk of blindness [1]. Eyes with high myopia that develop degenerative changes in the macula, optic nerve and peripheral retina are considered as having pathologic myopia, and are at the highest risk of developing potentially blinding complications such as retinal detachments, myopic choroidal neovascularization (CNV), myopic macular degeneration, foveoschisis, glaucoma, and cataract [2, 3]. Myopia has become a major public health issue because of its rapid increase of prevalence, especially in the East Asia, and its link to potential irreversible blindness.

Significant racial differences in the prevalence of myopia have been reported. The prevalence of high myopia is estimated to range between 2 and 5% in white populations and between 5 and 10% in Asian populations [4], while the prevalence of pathologic myopia is estimated to be ~1% in white populations and ~1–3% in Asians [5]. Regardless of these racial differences, there is evidence to suggest that the prevalence of myopia is increasing globally. A recent review estimated that 22.9% of the world population has myopia and 2.7% has high myopia in 2000, but by 2050, these figures will increase to 49.7% and 9.8%, respectively. In other words, almost 1 billion people will have high myopia [6], suggesting an alarming increase of prevalence globally.

Earlier age onset of myopia is a significant risk factor for high myopia in the future [7]. After adolescence, myopia progression gradually stabilizes for most individuals. The early onset of myopia in Asian schoolchildren is associated with longer duration to reach stability in refraction, and in some cases faster progression rate (-1 D per year) [8], which ultimately results in the higher prevalence of high myopia in Asian young adults, with risks to develop sequelae associated with high myopia and resulting in pathologic myopia [9]. Therefore, delaying myopia onset and retarding myopia progression in school-aged children is potentially the key to reduce high myopia later in life.

There are strong evidences to suggest environmental factors play a crucial role in the development of school age onset myopia [10], which include time spent outdoors [11], prolonged intense education [12], urbanization [10], near work [13], prenatal factors [14], and socioeconomic status [15]. Recently, outdoor activities and decreasing the duration of near work have been reported to be effective in delaying myopia onset [11, 16]. However, among the various interventions evaluated, atropine has been found to be one of the most consistently effective interventions in slowing down myopia progression[17, 18]. This review will cover recent understanding of pathogenesis of myopia, rationale, as well as clinical trial results of the use of atropine to retard myopia progression.

## Epidemiology of school myopia

Most individuals develop myopia at childhood, particularly during the school years; children with younger age at the onset of myopia tend to have greater myopia progression subsequently. Generally myopia at school age or juvenile-onset myopia often refers to the children who develop myopia in the primary school or early secondary school years, with the exclusion of the early onset forms of high myopia which are associated with strong familial inheritance [10]. Similar to the prevalence of myopia in adulthood, the prevalence and incidence rates of myopia in children varies among different areas and countries; in China and Taiwan, the annual incidence of myopia in 7–12-year-old children has been reported to be 8–18% [11, 19]. In contrast, a much lower annual incidence rate of 2.2% has been reported in children of 12-year-old in Australia [20].

Over the past decades, a number of reports have demonstrated that prevalence of myopia and high myopia has been increasing dramatically in schoolchildren, especially in the East Asia [21, 22]. For example, from 1983 to 2000 the prevalence of myopia in the 7-year olds increased from 5.8% to 21.0% in Taiwan [21]. In urban areas of East Asia, up to 80–90% of children completing secondary school are now myopic, and approximately one fifth of those has high myopia [23]. It is thought that environmental factors play a crucial role in this trend, as prevalence of school age onset myopia has remained low in the rural areas, such as in rural Mongolia, where the prevalence reported in 2006 was 5.8% [24].

European populations have had an estimated prevalence of 30.6% and the prevalence is steadily increasing [25, 26]. An increasing trend for the prevalence of myopia has also been observed in North America and Australia. According to a review in the United States, the prevalence of myopia in schoolchildren aged 12–17 years increased from 12.0% (between 1971 and 1972) to 31.2% (between 1999 and 2004) [27]. Another meta-analysis of population-based, cross-sectional studies of myopia prevalence in Western and Northern Europe demonstrated that there was a trend of higher myopia prevalence in the younger adults with a more recent birth year, of whom approximately half were affected [26]. Even in Australia, where prevalence of myopia appears to be lower than Europe and North America; it has been estimated that there may have been a four-fold increase in the prevalence of myopia over the last century [28].

## Current understanding of pathophysiology of myopia

The process of emmetropization is the adjustment of the length of the optical axis to the optical characteristics of the lens and cornea after the end of the second year of life. Myopization can be described as an overshooting of the process of emmetropization. In the first two years of life, the globe grows mostly spherically in all directions, increasing in sagittal diameter from about 17 mm at full-term birth to ~21 to 22 mm at the end of the second year of life. This eye growth is associated with an increase in the volume of the sclera and is thus probably accompanied by active formation of new scleral tissue [29]. Beyond the second year of life, further enlargement of the globe occurs predominantly in the axial direction, with 1 mm of axial elongation corresponding to a 0.5 mm increase in the horizontal and vertical diameters of the eye up to an axial length of 24 mm [30]. Beyond an axial length of 24 mm, the horizontal and vertical globe diameter increases by 0.2 mm or less for each mm of axial elongation. The axial elongation is associated with thinning of the choroid and, to a lower degree in relative terms, of the sclera. Choroidal and scleral thinning is most pronounced at the posterior pole and less marked at the equator [31]. The axial elongation is also associated with thinning of the retina and reduced density of the retinal pigment epithelium cells (RPE) in the retro-equatorial region, while retinal thickness and RPE cell density in the macular region and the thickness of Bruch’s membrane (BM) in any region are independent of axial length [32–34]. The axial elongation-associated increase in the fovea-optic disc distance is mainly due to the development and enlargement of parapapillary gamma zone defined as the BM free region around the optic disc [35, 36]. Subsequently, the length of BM in the macular region is not increased in axially elongated eyes, unless defects in BM in the macular region have developed [37]. The independence of the RPE cell density, retinal thickness, and length of the BM in the macular region fit with the observation that the best corrected visual acuity was independent of the axial length in axially elongated eyes without myopic maculopathy [38].

The process of emmetropization may occur in a feedback mechanism with an afferent, sensory part and an efferent part. Experimental studies in animals and clinical observations have suggested that the afferent sensory part may be located in the mid periphery of the fundus in the retro-equatorial region of the eye [39, 40]. This assumption is based on observations in animals that peripheral defocus leads to axial elongation of the eyes. In keeping with this hypothesis, patients with a congenital macular scar, e.g. due to a toxoplasmotic retinochoroiditis, usually do not develop axial elongation, while eyes with destruction of themed-peripheral retina, such as after laser photocoagulation for retinopathy of prematurity, can develop marked axial myopia. In contrast, eyes with retinopathy of prematurity treated by intravitreal application of anti-VEGF (vascular endothelial growth factor) drugs develop less axial myopia [41]. The notion of the mid-periphery fundus as the location of the sensory arm of the process of emmetropization is also supported by clinical trials on myopic children randomly assigned to wear single vision lenses or progressive addition lenses [42]. In contrast, understanding of the efferent arm of the proposed feedback mechanism has remained limited. The elusiveness includes the target tissue as well as the mode of communication between the afferent and efferent arms. A messenger molecule has been proposed to transfer the information from the afferent to the efferent arm. Proposed candidates include dopamine, levodopa, or a dopamine-like agonist that inhibited the axial elongation of occluded eyes with form-deprivation myopia in rabbits, guinea pigs or mice [43–45]. As a corollary, the intravitreal injection of apomorphine as a non-specific dopaminergic agonist resulted in an ocular growth inhibition in a lens-induced myopia model [46, 47]. However, contradictory findings have been reported in other animal models [48]. Another group of molecules proposed to be involved in the etiology of myopia were muscarinic antagonists. Studies revealed that pirenzepine, an anticholinergic agent with high muscarinic M1 receptor selectivity inhibited the axial elongation in guinea pigs, tree shrews, and in monkeys when applied intravitreally [49–51]. In guinea pigs, pirenzepine intraocularly applied increased the expression of tissue inhibitors of metalloproteinases (TIMP-2) and of tyrosine hydroxylase [51]. It fits with the results of clinical trials discussed later in this review, in which atropine applied topically in low concentrations of 0.01% was associated with a reduced progression of myopia in school-aged children. Another candidate molecule is the adenosine receptor antagonist, 7-methylxanthine [52].

The target tissue as the primary driver of the axial elongation has remained elusive so far. In many studies, the sclera, and in some investigations the choroid, have been considered to be primarily responsible for the myopic enlargement of the eye [53, 54]. The sclera as the primary driver of axial elongation does not fit however with the anatomical finding of a marked thinning of the choroid, most marked at the posterior pole and being in relative terms considerably more pronounced than the thinning of the sclera [25]. If the sclera was the primary tissue governing the axial length of the eye, one would expect a widening of the choroidal space. An alternative model could be to consider BM as the primary structure expanding posteriorly and compressing the choroid, most markedly at the posterior pole, and distending secondarily the sclera. This hypothesis is supported by several anatomical observations: (1) the volume of the sclera (and choroid) is not enlarged in axially elongated eyes, suggesting re-arrangement of available tissue without active formation of new tissue; (2) the thickness of BM is independent of the axial length; and (3) the goal of the process of emmetropization is the adaption of the length of the optical axis that ends at the photoreceptor outer segments. The first firm structure located closest to the photoreceptor outer segments is BM while the sclera is separated from the photoreceptor outer segments by the spongy choroid, the thickness of which additionally shows a diurnal variation. The notion of BM as the primary driver is supported by a recent study in which the biomechanical strength of BM in relationship to its thickness was about 50–100 times stronger as compared to the strength of the sclera (Girard, personal communication). This hypothesis also fits with the observation that the RPE cell density and retinal thickness in the fundus midperiphery decrease with longer axial length, perhaps due to the production of BM in that region leading to a mostly tube-like enlargement of the globe. If BM is the primary driver of axial elongation, the RPE producing BM as its basal membrane would be the target tissue. Interestingly, a recent experimental study on lens-induced myopia in young guinea pigs revealed that amphiregulin antibody if applied intravitreally was associated with a dose-dependent reduction in axial elongation [55]. The RPE has receptors for the epidermal growth factor with amphiregulin being a member of the epidermal growth factor family.

## Rationale for use of atropine

To date, atropine is the only medication that has been demonstrated to be consistently effective in slowing myopic progression [17, 18]. Once myopia has developed in a child, the rate of progression is estimated to be around −1 D per year in East Asians and around −0.5 D per year in Caucasians [8, 56]. Several years later, a significant proportion of these children will reach the definition of high myopia. Therefore, intervention to prevent myopia progression early on in myopic children is urgent and important. The higher concentrations of atropine such as 1% or 0.5% have been shown to be very effective in retarding myopia progression, but the high rate of photophobia side-effect (in up to 100%) has been associated with high dropout rate (16–58%) [57, 58]. In addition, there are concerns regarding potential long-term systemic or ocular side effects. Besides, rebound effect after atropine discontinuation has also been described, and is particularly notable in higher concentration of atropine. Recently, several publications from Asia have reported efficacy of 0.01% atropine in myopia control while having lower rates of side effects. As a result, there have been renewed interests in the clinical implementation of atropine for myopia control.

The exact mechanism of topical atropine is still not known, although the up- and downregulation of retinal and scleral muscarinic receptors with influence on the scleral matrix has been postulated [59, 60]. Moreover, atropine inhibits myopia induction in both mammalian and avian eyes [61, 62]. Different to the mammalian eye, the avian eye contains striated ciliary muscle innervated by nicotinic receptor rather than muscarinic receptors [63]. Therefore, atropine might have function at a relatively lower dose, through M1/M4 receptors in the retina, not via the accommodation system. On the other hand, a non-muscarinic and a direct influence of atropine on the scleral fibroblasts could also contribute to the effect [64].

## Review of clinical trials

### Refraction change of myopia

Anti-muscarinic agents was named as “the most likely effective treatment to slow myopia progression” in the Cochrane database systemic review of 2011. Among them, atropine is the most widely studied anti-muscarinic agents [17]. The randomized control trial in Taiwan, published by Yen et al. in 1989, reported that 1% atropine had better effect on controlling myopic progression in a year of follow-up visit when compared to 1% cyclopentolate and placebo [58]. The mean myopia progression was −0.22 ± 0.54 D per year in eyes receiving 1% atropine, which was lower compared to 1% cyclopentolate (−0.58 ± 0.49 D per year) or placebo groups (−0.91 ± 0.58 D per year). Shin et al. later published another randomized control trial [8]. Children aged 6–13 years received tropicamide and served as the control group, in comparison with those who had 0.5, 0.25, or 0.1% of atropine. After 2 years of follow-up, all the atropine groups showed a positive effect on reducing myopic progression. Sixty-one percent of children in the 0.5% atropine group had cessation of myopic progression, while 4% had rapid progression. A lower proportion of eyes had cessation of myopia progression was observed in the control compared with the atropine groups (49%, 42% and 8% in the 0.25% atropine, 0.1% atropine and control group, respectively). Concurrently, a higher percentage of children with rapid progression was observed in the control group compare with the atropine treated children (17%, 33% and 44% in the 0.25% atropine, 0.1% atropine, and control group, respectively).

Atropine for the treatment of childhood myopia (ATOM) 1 study enrolled 400 school-aged children with myopia (spherical equivalent −1.00 to −6.00 D) and low astigmatism (≤1.5 D) in a double-masked trial in which, half of the enrolled children received 1% atropine in one eye nightly, and the other half received vehicle eye drops as the placebo [65]. After 2 years, the mean progression of myopia was significant lower in the 1% atropine group (−0.28 ± 0.92 D), compared to the control group (−1.20 ± 0.69 D). Atropine was stopped after 2 years and the children were observed for a further 12 months. During this “wash-out year”, myopic rebound was observed, which was more marked in the atropine group (−1.14 ± 0.80 D) than the placebo group (−0.38 ± 0.39 D) [66]. Despite the rebound, the overall myopic progression was still less for the eyes treated with atropine than placebo, over the 3-year period. Ocular side effects included photophobia, glare, and loss of accommodation. The use of bifocal or multifocal glasses could be used to relieve the symptom of blurred vision at near, Use of sun glasses or photochromic tinted lenses helped to relieve the symptom of photophobia and minimize the risks of cataract and retinal phototoxicity by excess exposure of Ultraviolet light irradiating into the eye. In addition, the cycloplegic effect was found to recover upon cessation despite long-term chronic use of atropine eye drops [66].

ATOM 2 study recruited another 400 children in Singapore and randomized them in 2:2:1 ratio to receive 0.5%, 0.1%, and 0.01% atropine per night for 2 years, in order to clarify if atropine at lower concentration could have similar efficacy as 1% atropine dosing [67]. The mean progression in the first 24 month (phase 1) was −0.30 ± 0.60 D, −0.38 ± 0.60 D, and −0.49 ± 0.63 D in the 0.5%, 0.1%, and 0.01% atropine arms respectively (0.01% vs. 0.5%, p = 0.02; between other concentrations: p > 0.05). Atropine was stopped at the end of 2 years, and all participants were monitored for a year (phase 2) [68]. During this ‘wash-out period’, rebound progression of myopia was more prominent in the 0.5% atropine group (−0.87 ± 0.52 D), compared to the 0.1% (−0.68 ± 0.45 D) and 0.01% group (−0.28 ± 0.33 D, p < 0.001). The overall progression of myopia over the 36-month period was significant lowest in the 0.01% atropine group (−0.72 ± 0.72 D) followed by the 0.1% atropine group (−1.04 ± 0.83 D) and the highest progression was observed in children that were treated with 0.5% atropine group (−1.15 ± 0.81 D) (p = 0.0002). 192 children who had rapid progression of myopia (defined as more than -0.5D/ year) within the washout year of phase 2 went on to restart the 0.01% atropine for 2 future years (phase 3) [69]. At the end of this 5-year clinical trial, the overall progression of spherical equivalence myopia in the 0.01% atropine group (−1.38 ± 0.98 D) was significantly less compared to the 0.1% and 0.5% atropine 1.83 ± 1.16 D and −1.98 ± 1.10 D; *p* < 0.05).

### Change in axial length

Excessive elongation of the globe is believed to contribute significantly to the degenerative changes of pathologic myopia [70], the biometric characteristics of myopia control is considered an important area to study. In 2001, 188 school-aged participants were treated with 0.5% atropine plus multi-focal spectacles vs. multi-focal glasses alone, or single-vision glasses alone, in a double-blind randomized control trial [71]. After regularly followed up at a single center in Taiwan for 18 months, the increase in the axial length in the atropine plus multi-focal glasses group was significantly less than the other two groups (*p* = 0.0001).

Atropine, particularly in higher concentrations, has been shown to have a positive effect in reducing the elongation of axial length in myopic eyes. The mean increase in axial length in ATOM 1 study was 0.02 ± 0.35 mm in the 1% atropine group, after 24 months of follow-up [65]. During the same period, a significantly more marked increase in axial length (0.38 ± 0.38 mm, *p* < 0.001) was observed in the placebo group, as well as the untreated fellow eyes of atropine group. This effect on the axial length in the atropine group was maintained until the end of the wash-out period. At the end of the third year, mean axial length increase was 0.29 ± 0.37 mm in the 1% atropine group, while the placebo-treated eyes experienced a mean increase of 0.52 ± 0.45 mm (*p* < 0.0001) [66]. To further understand the biometrical changes during atropine treatment, 313 subjects in ATOM 1 study completed biometric measurements including cycloplegic autorefraction, corneal curvature, anterior chamber depth, lens thickness, vitreous chamber depth, and axial length [72]. Eyes with myopic rebound at the end of the 3-year clinical trial were accompanied by a prominent increase in vitreous chamber depth and axial length (both had a *p* value < 0.001). It suggested that the main effect of atropine in tempering myopic progression was by slowing the growth of vitreous chamber depth and axial length.

In the ATOM 2 study, the mean change in axial length after 2 years was 0.27 ± 0.25, 0.28 ± 0.28, and 0.41 ± 0.32 mm in the 0.5%, 0.1%, and 0.01% atropine treated group, respectively (*p* < 0.001when comparing 0.01% with 0.1% or 0.5%). However, the situation reversed after the 1-year washout period. At the end of the third year, eyes in the 0.01% atropine group experienced the least increase in axial length (0.19 ± 0.13 mm), compared to eyes in the 0.5% and 0.1% atropine group (0.35 ± 0.20 and 0.33 ± 0.18 mm respectively, *p* < 0.001). A slower increase in axial length continued to be observed in the 0.01% atropine group during phase 3 of the study (0.19 ± 0.18 mm), in comparison with 0.1% atropine (0.24 ± 0.21 mm, *p* = 0.042) and 0.5% atropine (0.26 ± 0.23 mm; *p* = 0.013) groups. However, at the end of the 5-year study, there was no significant difference in axial length increase between the three groups (0.75 ± 0.48, 0.85 ± 0.83, 0.87 ± 0.49 mm; *p* = 0.185) [69].

### Response rate

Shih et al. found 10.6% of children did not respond to atropine 0.5% [71]. In another study, progressors (defined as >1 D increase of myopia /year) was found in 4% of the 0.5% atropine group, 17% of the 0.25% atropine group and 33% of the 0.1% atropine group, in comparison to 44% in control group [8]. In a retrospective cohort study, Wu et al. found 45% of children were “poor responders” to 0.05% atropine, and they continued to progress by >0.5 D over 6 months [73]. These poor responders were switched to 0.1% atropine and over the 4.5 years follow-up, around 20% progressed further by >0.5 D per year, although this rate was much lower than those without treatment (100% progressed by >0.5 D per year). Despite the encouraging overall results in ATOM 1 study, not all the participants had good responses to atropine. 12% of children treated with atropine 1% at 1 year continued to progress by >−0.5 D per year [74]. These “progressors” were more likely to be younger, more myopic or have 2 myopic parents. In ATOM 2, 4.3%, 6.4%, and 9.3% of children in the 0.5%, 0.1%, and 0.01% group, respectively, had myopia progression ≥−1.5 D over the initial 2-year of active treatment.

### Side effects

Systemic side effects in the ocular use of atropine is uncommon, such as dry mouth, face flush, headache, increased blood pressure, constipation, difficulty in micturition, and central nervous system disturbances. The most frequent ocular side effects with atropine eye drops include photophobia, blurriness of near vision, and local allergic response. Among them, photophobia is the most common and its incidence is positively correlated with the concentration of atropine. All of the patients who received 1% atropine in the study of Yen et al. reported photophobia, and this was described as the major reason that led to over a half of subjects dropping out of the study [58]. In contrast, photophobia was reported in only 22% and 7% of participants who received 0.5% and 0.25% atropine, respectively. None of the participants in the 0.1% atropine group reported significant photophobia [8]. Similarly, photophobia was uncommon in children who received 0.01% atropine in ATOM 2 study, and only 7% of subjects requested photochromatic lenses.

Among the 34 participants (17%) who withdrew from ATOM 1 study, the reasons were hypersensitivity, glare, and poor near-visual acuity. As for ATOM 2, 4.1% children in 0.1% and 0.5% atropine group reported allergic conjunctivitis [7]. Reduction of near visual acuity was reported in the 0.1% and 0.5% groups, but completely recover by 26 months. Rarely, glaucoma may be induced by atropine. The incidence is as low as 1 in 20,000 [75]. One study reported 621 children treated with atropine for 3 year and none found ocular hypertension [76].

### Clinical trials from non-Asian populations

Since the prevalence of myopia is much higher in Asian than in other areas, it is not surprising that majority of randomized trials in myopia control have been conducted in Asia. Nonetheless, several cohort studies about myopia in non-Asian population had been published. These studies are also important in addressing initial concerns for potential ocular side effects, particularly photophobia in patients with lightly pigment and light-colored iris.

The effectiveness of 1% atropine in myopic control had been demonstrated by Brodstein et al., in 1984, and Kennedy et al., in 2000 [77, 78]. They both recruited more than 200 children and teenagers in the United States, with the mean follow-up of around 4 years. Another smaller study in United States, included 15 myopic children treated with 1% atropine and the other 15 children as control. The mean annual myopic progression was decreased to 0.05 D compared to 0.84 D in the controls [79]. 0.5% atropine once daily was used in a study based in Rotterdam with a sample size of 77 children with progressive myopia (defined as spherical equivalent (SE) ≤ −3D and SE progression rate ≥1 D per year under cycloplegic conditions) [80]. The aim of this study was to determine whether 0.5% atropine was effective at slowing the myopia progression, as well as to study the adherence to therapy in a setting outside Asians. Half (50.6%) of the children were already highly myopic (SE > −6 D). Of adverse events reported, photophobia was common (72%), followed by reading problems (38%), and headaches (22%). More than half (60%; 36/60) of the children adhered to therapy. However, 17 stopped treatment, of whom 11 (64.7%) discontinued treatment within the first month. The progression of myopia was −1.0 D per year before treatment diminished substantially to −0.1 D per year after 1 year treatment in the continued treatment group. Children who completed the 12-month trial benefited more than those who stopped prematurely. The study concluded that atropine at 0.5% can be effective for treating progressive myopia in Europe and suggested that intervention with atropine could work irrespective of ethnicity.

Clark et al. performed a study using low concentration atropine on 60 school-aged children in California [56], and reported slowing of myopia progression rate in 0.01% atropine-treated eyes (−0.1 ± 0.6 D per year) compared to control eyes (−0.6 ± 0.4 D per year, *p* = 0.001). Only three subjects in the atropine group complained of intermittent blurred vision or light sensitivity. None discontinued the treatment due to the symptoms. A Spanish study used 0.01% atropine in 400 eyes of 200 children aged between age 9 and 12 years, randomized to treatment vs. no treatment [81]. The mean annual myopia progression of the treated group was −0.14 ± 0.35 D per year vs. −0.65 ± 0.54 D per year in the control group without treatment over a 5-year follow-up period. The authors concluded that 0.01% atropine can slow myopia progression. Only 2% of patients stop treatment due to side effects.

To determine the highest dose of atropine that can be well tolerated, 12 subjects, ages from 8 to 16 years old, were evaluated by Cooper et al. [82]. They found that accommodation becomes affected in atropine above 0.02%. In keeping with this report, 14 Caucasian participants aged above 18 years were given 0.01% atropine for 5 sequential days [83]. The treatment was well tolerated by all participants and none reported problems in near or distant vision.

## Clinical implementation

Based on the results of randomized controlled trials, atropine treatment has been implemented in clinical practice in some countries, mostly in Asia. Currently, the clinical management of myopia in Europe is predominantly focused on refractive correction, as data from studies on European populations remain limited.

### Initial assessment

Regardless of whether atropine treatment is available, the refractive error should be determined accurately in all children referred for refractive error at their first consultation. Cycloplegic refraction is the gold standard for clinical practice or research [84]. The accommodation amplitude could be >10 D in a 10-year-old child [85]. Due to this large range of accommodation, pseudo-myopia is commonly noted if only auto-refractometry examination is used. Short and mid-duration cycloplegic agents include tropicamide or cyclopentolate and long-duration cycloplegic agents such as atropine could be used for cycloplegic refraction in children [86, 87].

After cycloplegic refraction, spherical equivalent refractive error (SER) could be categorized as hyperopia, pre-myopia, and myopia. The definition of hyperopia is the SER > +0.5 D and the definition of myopia is SER ≤ −0.5 D. The definition of pre-myopia is SER ≤ +0.5 D and > -0.5 D [88].

Hyperopic children should be educated regarding good eye care, including encouragement for outdoor activities around 2 h every day and near work breaks [89–91]. Regular follow-up with cycloplegic refraction every half or 1 year to monitor the speed of myopic refraction shift until the end of adolescence is suggested. In the United Kingdom, the National Health Service offers optical vouchers which cover annual free eye test and spectacles for all children up to age 16 (and age 19 if in full time education). The eye test can be performed more frequently if there are new symptoms.

For pre-myopic children, hyperopic refraction <+0.75 noted during the elementary school period has been reported to be a risk for subsequent myopia onset [92]. The overall evidence for the benefit of atropine treatment in the pre-myopic children is still limited. Fang et al. reported 24 of 50 pre-myopic children received 0.025% atropine at bedtime decreased the onset of myopia from 54% to 21% compared to the control group after 1 year [88]. However, larger studies with longer follow-up are needed to determine whether atropine should also be started in all pre-myopic children. Similarly the optimal dose will need to be evaluated. Monitoring the myopia shift every 3–6 months according to child’s age and parent’s myopia history is recommended. In the United Kingdom, children aged between 4- and 5-year old have visual screening performed at school, predominantly by orthoptists. Those that found to have suboptimal vision, amblyopia or abnormal eye movements are being monitored and refract accordingly in their local pediatric eye department. The older children, without ocular disease, are being monitored 6–12 monthly, in the community, outside the setting of hospital eye service.

### Commencing atropine treatment

For myopic children, atropine treatment can be offered with the aim to slow down myopia progression. Before starting, the treatment aim and procedure, potential side effects, success criteria and rate should be discussed. It is important for parents and children to understand that atropine treatment works to slow down myopia progression but does not improve the vision as with orthokeratology. However, the risks associated with atropine treatment are relatively low and the benefits may last long term. The course of treatment is expected to be a minimum of 2 years initially, after which the child should be monitored to keep the low myopia status until the end of adolescence. Concurrent to atropine treatment, outdoor activities should continue to be encouraged [93]. The child’s age, baseline refractive error, any evidence of recent progression, and refractive error of parents may help to predict the likelihood of progression. Starting treatment with the lowest concentration, such as 0.01% atropine, would be preferable as this is associated with the least ocular side effects. The dosing frequency is once daily at bed time. A small hyperopic shift is often noted 2–3 weeks after initiation of atropine, which may result from backward relaxation of ciliary body and lens zonular fiber becoming taut. Therefore, record of both the baseline without treatment and the post-atropine baseline refraction after 2–4 weeks later is useful. Thereafter, follow-up every 3 months with the cycloplegic refraction is recommended.

### Assessment after starting atropine treatment

During the period of atropine treatment, the appropriate distance glasses should be prescribed if the child has difficulty in far vision, such as looking at blackboard in classroom or watching TV. It may be useful to explicitly explain to patients that while glasses improve vision, they have no clinical significant effect on myopia control [94, 95]. However, it is recommended that the child removes the distance glasses during near work, as distance glasses could induce hyeropic defocus during near-work and is believed to contribute to further myopia progression [96, 97]. In addition, distance glasses can aggravate symptom of near-blur in children on atropine treatment. Children who experience near-blurred symptom can also be offered bifocal or multi-focal glasses. Hat, photochromic, or sunglasses are recommended during outdoor activities to prevent photophobia symptoms. During the annual review, it is good clinical practice to check for side effects such as dry eye, allergic conjunctivitis, flushing, headache, heart, and urinary symptoms. The standard examinations include non-contact axial length, funduscopy to screen for myopic related peripheral retinal degeneration, e.g., lattice or breaks, and intraocular pressure measurement.

**Fig. 1:**
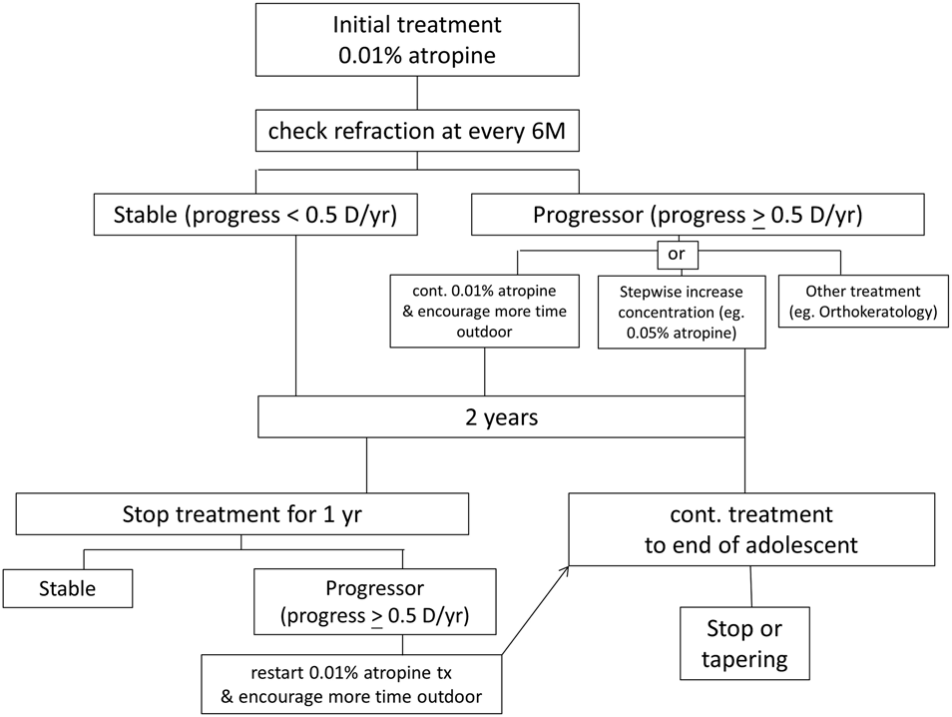
The proposed strategy of atropine treatment for myopia control in clinical implementation

If myopia continues to progress by ≥0.5 D in Asian children after 6 months or in Caucasian children after 1 year, it indicates the efficacy of the current treatment dosing is probably not adequate. The management strategy in these suboptimal responders remains unclear. Alternative strategies include increasing the concentration of atropine, or continue the same concentration of atropine combined with more outdoors time, or, change to a different treatment modality, such as orthokeratology (Fig. 1). The evidence for better result with higher doses of atropine is, however, limited [69]. The higher rate of side effects and potentially higher discontinuation rate should also be considered. Wu et al. had reported the stepwise method for the myopic long-term control [73]. They recommended stepwise increase in the concentration of atropine according the effect of myopia control.

While most studies have reported active treatment period of 1–2 years, the optimal length of treatment is not known. One strategy is to adopt the ATOM 2 study approach with 2 years of initial treatment, followed by withholding treatment for 1 year, during which time any further progression is monitored. Children who progress after stopping treatment can be offered further treatment. Alternatively, some centers in Taiwan adopt the continuous treatment till late adolescent (around 15–18 years old), as myopia progression is known to slow down in the late adolescent period [98, 99]. Some investigators suggest tapering instead of abrupt stop to prevent possible rebound effect; however this has not been studied in detail.

### A European perspective

In Europe, atropine eye drops 1% are commonly used in the treatment of amblyopia, but the adoption of atropine treatment for myopia retardation has been less widely practice than in Asia. This is likely due to a combination of factors, e.g., limitation of data from European studies, lack of availability of licensed preparation of atropine at ultra-low concentration, as well as cultural differences and attitudes towards side effects. Although there are good evidence to suggest atropine could be effective, more research is needed in European populations to propel the clinical application. In addition, there are logistic challenges. In the United Kingdom, the 0.01% atropine eye drop is not available as a licensed product. The “do-it-yourself” or self-dilution method is not endorsed, due to inaccurate dosing and potential issues with infection control. There are some motivated families manage to access the 0.01% atropine eye drops from south-east Asian countries, through personal connections.

There is also the anticipated barrier for children and their families to use atropine in the European population due to less acute awareness of myopic-related ocular complications and fundamental cultural differences, compared to the Asian populations. Parents and children may be more focused on the immediate potential side effects from atropine, particularly in those with lighter iris colors. The differences in the appearance of their pupil size compared with pre-treatment level could potentially attract unwanted attention from their peer groups, which could be interpreted as teasing or bullying. The negative psychosocial impact of appearance of more dilated pupils and possible photosensitivity could lead to a premature cessation of therapy.

The 0.01% atropine eye drops is not being used as a standard clinical treatment for myopia in the National Health Service at present, but its popularity is likely to surge when a licensed product become available.

In conclusion, results from research have demonstrated low concentration of atropine is useful in retarding myopia progression in a certain proportion of myopic schoolchildren. Atropine treatment has now been incorporated into clinical practice in some Asian countries. However, for optimal results, the motivation of parent and children is important, and long-term compliance and adherence with atropine treatment cannot be over-emphasized. Education regarding the consequences of high myopia and sharing the effect of myopia control to children and parents at each visit are helpful strategies to keep them motivated during the course of treatment. Individualized treatment protocol of atropine starting from low concentration seems practical. On top of atropine, good eye-care habits, enhancement of time outdoors and limiting near-work load should also not be overlooked. Though low-dose atropine treatment is promising in myopia control, there are still remaining areas of uncertainty such as treatment strategy and targeting population. Although the current prevalence of myopia in Europe is not as high as in Asia, the prevalence of myopia is steadily rising in Europe and US as well. The clinical and economic burden will become significant with time, therefore further research on myopia prevention in European populations is important.
